# Proteolytic chemokine cleavage as a regulator of lymphocytic infiltration in solid tumors

**DOI:** 10.1007/s10555-019-09807-3

**Published:** 2019-09-03

**Authors:** Holger Bronger, Viktor Magdolen, Peter Goettig, Tobias Dreyer

**Affiliations:** 1 Clinical Research Unit, Department of Obstetrics and Gynecology, Technical University of Munich, Ismaninger Straße 22, D-81675 Munich, Germany; 2 Division of Structural Biology, Department of Biosciences, University of Salzburg, Salzburg, Austria

**Keywords:** Chemokines, Proteases, Tumor-infiltrating lymphocytes, CXCR3, CX3CR1, Cathepsins, Matrix metalloproteinases, Dipeptiyl peptidase 4

## Abstract

In the past decade, immune-based therapies such as monoclonal antibodies against tumor epitopes or immune checkpoint inhibitors have become an integral part of contemporary cancer treatment in many entities. However, a fundamental prerequisite for the success of such therapies is a sufficient trafficking of tumor-infiltrating lymphocytes into the tumor microenvironment. This infiltration is facilitated by chemokines, a group of about 50 small proteins capable of chemotactically guiding leukocytes. Proteolytic inactivation of chemokines leading to an impaired infiltration of immune effector cells appears to be an efficient immune escape mechanism of solid cancers.

The CXCR3 and CX3CR1 chemokine receptor ligands CXCL9-11 and CX3CL1, respectively, are mainly responsible for the tumor-suppressive lymphocytic infiltration into the tumor micromilieu. Their structure explains the biochemical basis of their proteolytic cleavage, while *in vivo* data from mouse models and patient samples shed light on the corresponding processes in cancer. The emerging roles of proteases, e.g., matrix metalloproteinases, cathepsins, and dipeptidyl peptidase 4, in chemokine inactivation define new resistance mechanisms against immunotherapies and identify attractive new targets to enhance immune intervention in cancer.

## Introduction

Although it was postulated more than 100 years ago that the immune system can fight established solid tumors, only in the past two decades substantial progress has been made towards a better understanding of the interaction between the different subtypes of immune cells with tumor cells and their environment [1, 2]. Starting point for many of these studies was the clinical observation that the number of tumor-infiltrating lymphocytes (TILs) is a strong and robust prognostic marker across various tumor entities, such as breast, colon, or ovarian cancer [3–5]. Further studies have dissected the different subpopulations of TILs delineating either their tumor-suppressive (e.g., cytotoxic T cells, natural killer cells) or tumor-promoting functions (e.g., regulatory T cells) [6, 7]. Deciphering the immune cell-tumor cell interactions has prompted the development of new cancer immunotherapies, such as monoclonal antibody therapy against tumor antigens, immune checkpoint inhibition, adoptive T cell transfer, or various vaccination strategies [8]. Especially in highly immunogenic tumors, these immunotherapies have led to unprecedented improvements in survival and quality of life of cancer patients. Examples include the HER2-directed therapies in breast cancer or the immune checkpoint inhibitors in melanoma or non-small cell lung cancer [9, 10]. Moreover, there is ample evidence that classic chemotherapies also work *via* stimulation of an anti-tumor immune response in addition to their cytotoxic effects on the individual cancer cells [11].

However, a fundamental prerequisite for the success of all of these approaches is sufficient trafficking of the respective immune effector cells into the tumor microenvironment [12, 13]. This renders TILs or TIL subpopulations not only prognostic, but also feasible predictive biomarkers for the response to these therapies [7, 14]. In addition, it raises one of the most urgent questions in contemporary cancer immuno-oncology: how can these immune cells be efficiently recruited into the tumor? The ultimate goal is to transform a “cold” into a “hot” tumor [15].

The recruitment of immune cells into solid tumors is mediated by chemokines, a family of about 50 small proteins capable of chemotactically facilitating leukocyte migration [16]. Different chemokines can either attract tumor-suppressive or tumor-promoting leukocytes, and, thus, the intratumoral chemokine milieu is a strong determinant of the intratumoral immune milieu [17]. Besides their chemotactic function, chemokines also participate in activation or inactivation of immune cells.

Many chemokines can be posttranslationally modified by proteolytic cleavage which either activates or destroys their chemotactic function [18]. Expression of the corresponding proteases may thus significantly influence the modulation of the immune milieu and the anti-tumor immune response. Therefore, proteolytic inactivation of tumor-suppressive chemokines represents a potent immune escape mechanism of solid tumors [19]. Conversely, inhibition of these proteases might be an attractive adjuvant to immunotherapies, such as immune checkpoint inhibitors, whose function depends on the activity and presence of specialized chemokines [20].

**Fig. 1:**
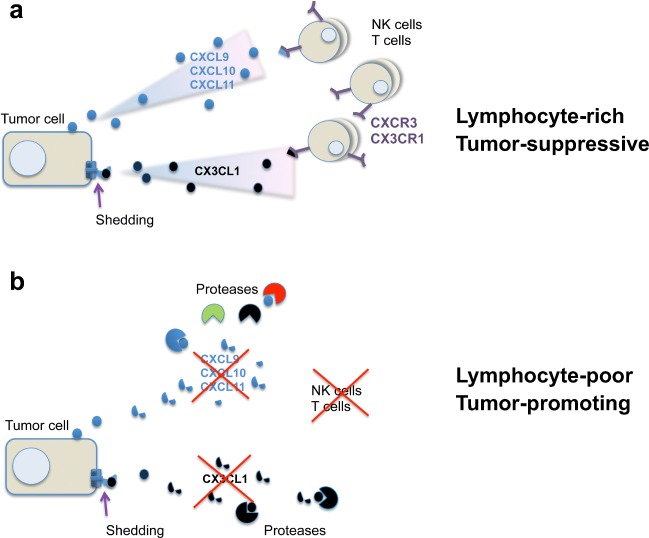
Inactivation of chemokine-mediated lymphocyte infiltration by proteolytic cleavage. **A** Secretion of the chemokines CXCL9, CXCL10, and CXCL11 as well as shedding of CX3CL1/fractalkine leads to chemotactic recruitment of tumor-suppressive lymphocytes to the tumor site. **B** Chemokine-targeted cleavage by proteases, such as MMPs, cathepsins, and DPPs, impairs lymphocytic infiltration, leading to reduced amounts of immune cells in the tumor microenvironment

In the following, the concept of chemokine cleavage as a modulator of the anti-tumor response will be discussed. The focus will be on the chemokines that are most notably known for their ability to recruit tumor-suppressive lymphocytes such as cytotoxic T cells (CTLs) or natural killer (NK) cells into solid tumors **(**Fig. 1**)**. These chemokines are the CXCR3 receptor ligands CXCL9, CXCL10, and CXCL11 as well as the CX3CR1 ligand CX3CL1, also named fractalkine [17, 21].

## Chemokines

Chemokines or chemotactic cytokines make up the largest family of cytokines. With 48 members expressed in human tissue, they represent a structurally and functionally related group of low molecular weight proteins, which controls chemotactic recruitment of specific lymphocyte subtypes in a tissue- and time-dependent manner [22]. The distribution of immune cells not only plays a crucial role in the innate and adaptive immune response in case of inflammation or associated disease [23–27], but also supports tissue homeostasis [28] and angiogenesis [29, 30]. According to their major function chemokines are classified into two groups [25]: Homeostatic chemokines are constitutively expressed in order to initiate a proper immune response and to define the composition and organization of tissue-resident, blood-derived cells such as macrophages and dendritic cells [16]. On the contrary, inflammatory chemokines are mainly inducible and are strongly upregulated upon demand [31]. It is of note that this classification is not strict as there are chemokines that can contribute to both groups.

The conventional chemokine signaling is thought to be transduced through a complementary receptor network consisting of transmembrane G protein-coupled receptors [32]. However, also receptor-independent modulation of signaling has been proposed. Factors that can influence chemokine signaling are non-canonical receptors such as the atypical chemokine receptors 1-4, mRNA instability, alternative splicing, or glycosaminoglycan (GAG) binding [33–35]. Furthermore, regulation is even more complex due to considerable promiscuity between chemokines and their receptors, i.e., several ligands may bind to the same receptor and *vice versa* a single ligand can activate different receptors [16]. Moreover, chemokine receptors are involved in several signaling cascades which can be differentially activated by either distinct ligands or *via* oligomerization of the same ligand [36, 37].

**Fig. 2:**
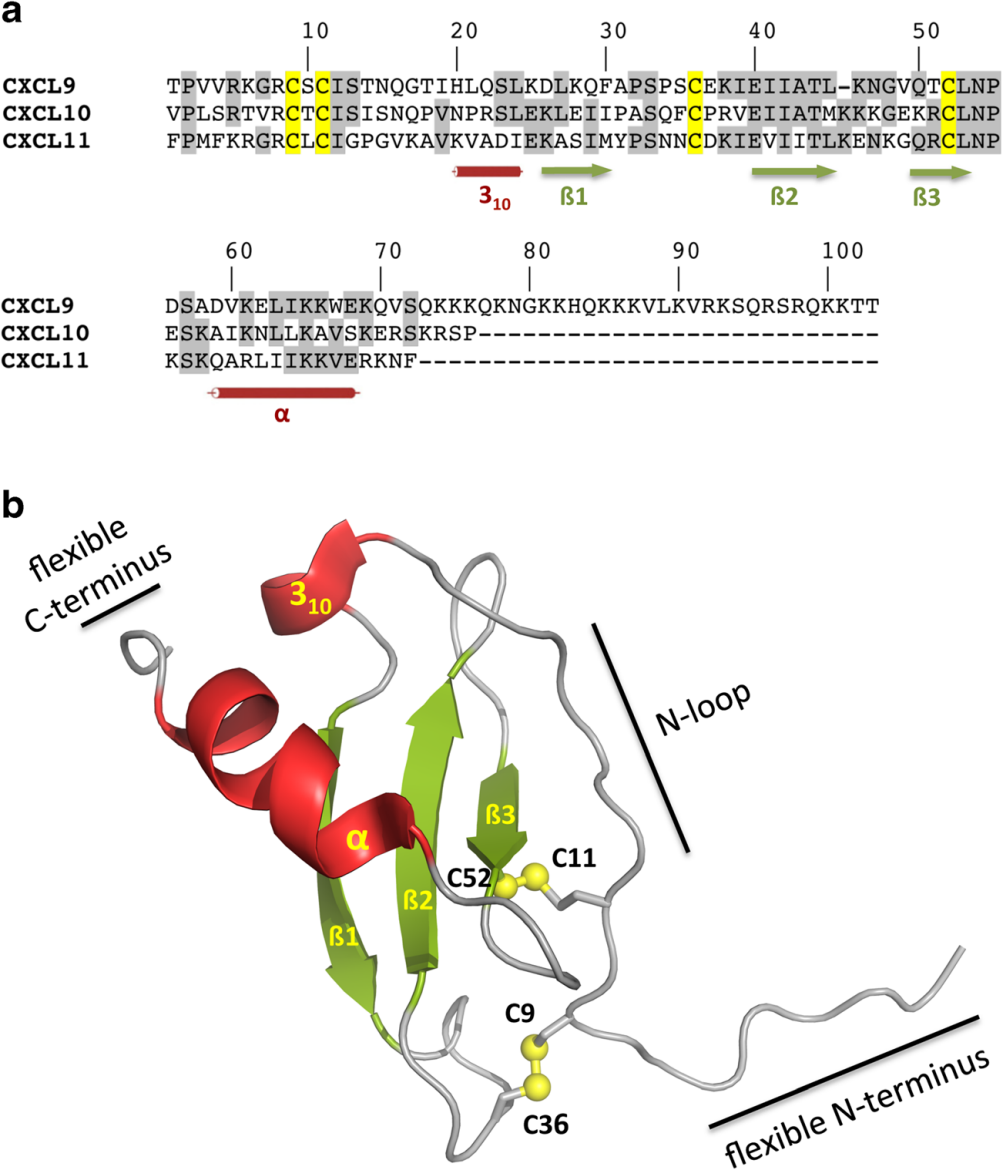
Primary and tertiary structure of CXC chemokines. **A** Sequence alignment of human CXCL9, CXCL10, and CXCL11. Secondary structure elements are indicated by green arrows (ß-strands) and red cylinders (α-helix and 3_10_-helix). Cysteine residues are indicated in yellow, whereby Cys^9^ forms a disulfide bridge with Cys^36^ and Cys^11^ with Cys^52^, respectively. The numbering is derived from CXCL9. **B** Ribbon plot of model 1 from the NMR structure of CXCL11 (PDB code 1RJT). A flexible unstructured N-terminus is followed by the N-loop, a 3_10_-helix (red), and the central three-stranded anti-parallel ß-sheet (green), which is stabilized by two disulfide bridges, depicted as yellow spheres. Near the C-terminus an α-helix (red) is present, which packs onto the ß-sheet structure, followed by a flexible C-terminus

The structure of chemokines is conserved across subfamilies, in most cases built around four conserved cysteine residues, except the XC chemokines which lack two of these four cysteines [38]. The organization of the first cysteine residues in the N-terminal part of the chemokines divides them into four different groups: CXC, CC, XC, and CX3C, with X representing any amino acid [39]. The cysteines form one or two disulfide bridges, which stabilize a central three-stranded anti-parallel ß-sheet with a rigid loop structure (Fig. 2). This central core is preceded by a flexible unstructured N-terminus. Both the flexible N-terminus and the following rigid N-loop, which often is C-terminally limited by a 3_10_-helix, within the globular core are functional domains of the chemokine in terms of receptor binding and activation. Originally, a two-site/two-step model had been proposed, in which firstly the N-terminus of the receptor interacts with the N-loop of the chemokine and, subsequently, the unstructured N-terminus of the chemokine can enter the binding pocket of the receptor resulting in receptor activation and transmembrane signaling [40]. Recent results indicate that this model may have to be extended to a three step model, in which step 1 of the original model, binding of the chemokine to the receptor, is divided into an initial low-affinity, rather unspecific binding which is then followed by the specific high-affinity binding of the chemokine to the receptor [41]. C-terminal of the chemokine core, an α-helix is present which packs onto the ß-sheet structure. The glycosaminoglycan (GAG) binding site of the chemokines is located within the β-sheet and C-terminus [42].

## The CXCR3 chemokine system

The CXC chemokines can be further divided according to the presence or absence of a highly conserved Glu-Leu-Arg (ELR) motif, which is shared by CXCL1-3 and CXCL5-8. Among the chemokines that lack the ELR motif (CXCL4, CXCL4L1, and CXCL9-14), most of them interact with the CXCR3 receptor [43]. The CXCR3 receptor is a 368 amino acid (aa) seven-transmembrane G protein coupled receptor with three isoforms. The originally identified, canonical CXCR3 receptor, named CXCR3-A, is bound by the ligands CXCL9, CXCL10 and CXCL11 and, upon activation, promotes chemotaxis, invasion, proliferation, and cell survival [44]. The first identified splice variant of CXCR3 was called CXCR3-B and interacts with CXCL4 and CXCL4L1 in addition to the classical ligands. This receptor isoform does not trigger chemotaxis but instead growth suppression, angiostasis, and apoptosis. The most recently discovered CXCR3 isoform, CXCR3-alt, only accepts CXCL11 as ligand [44, 45].

CXCR3 receptor expressing cells include, among others, regulatory T cells, CD4^+^ and CD8^+^ T cells, dendritic cells, NK cells, and NKT cells [21, 46]. CXCR3 ligand expression can be detected in endothelial cells, keratinocytes, and fibroblasts. Also, immune cells like T cells and monocytes are capable of secreting those chemokines. Especially CXCL9 and CXCL11 are secreted by peripheral blood monocytes and macrophages [22].

CXCL4 and CXCL4L1 are described as platelet-related agonists, whereas CXCL9, CXCL10, and CXCL11 expression is strongly inducible by interferons (IFN). All of the latter cytokines can be induced by IFN-γ, but CXCL11 is the only one being also induced by IFN-α [47]. CXCL11 show the highest binding affinity towards CXCR3 followed by CXCL10 and CXCL9. Among these three chemokines, CXCL9 shows the weakest activation upon receptor binding [22]. Interestingly, competition with CXCL11 receptor binding by CXCL9 or CXCL10 is always incomplete. Furthermore, CXCL11—in contrast to CXCL10—does also bind to CXCR3, when the receptor is uncoupled from G protein-dependent signaling [48]. Receptor activation by its three ligands also results in different effects. CXCL11 was described to be the most potent inducer of receptor internalization, whereas CXCL9 and CXCL10 mainly induce chemotaxis as well as Ca^2+^ influx [49].

Besides induction of migration and Ca^2+^ influx, ligand/receptor interaction can also lead to downstream phosphorylation of target proteins, e.g., transcription factors of the STAT family. CXCL9 and CXCL10 lead to the phosphorylation of STAT1, STAT4, and STAT5 and subsequently to an activation of T-bet and RORyT, two differentiation regulators, resulting in polarization of CD4-positive T cells towards the Th1 and Th17 effector lineage. Contrariwise, CXCL11 binding induces phosphorylation of STAT3 and STAT6, which leads to a regulatory phenotype of CD4-positive T cells (Th2 or Tr1) [50–52].

Thus, together with the varying susceptibility to post-translational modification, differences concerning ligand-receptor interaction and GAG binding, and the fact that CXCL9, CXCL10, and CXCL11 display not completely overlapping functions, a complex regulatory network is formed affecting diverse biological functions.

## The CX3CR1/CX3CL1 chemokine system

Cells expressing the seven-transmembrane receptor CX3CR1 (355 aa) are monocytes, macrophages, NK cells, lymphocytes, and dendritic cells [53]. Its ligand, CX3CL1, also named fractalkine, is the only member of the CX3C chemokine family. It is expressed as a membrane-bound protein, but is also found in soluble forms [54, 55]. The 317 aa long extracellular domain encompasses a stalk formed by mucin-like domains with a chemokine domain on top. This N-terminal extracellular part is followed by a transmembrane domain and a short C-terminal cytoplasmic tail (34 aa). The soluble forms of CX3CL1 carrying the mucin-like stalk [56] and possibly also the cytokine domain only [57] provide chemotactic recruitment of CX3CR1-positive cells. As a membrane-bound protein, CX3CL1 provides adhesive capacity and helps to stabilize the interaction between CX3CL1-expressing cells and cells expressing the corresponding receptor CX3CR1, respectively [58]. CX3CL1/CX3CR1-mediated adhesion can be further enhanced by synergistic interaction with other adhesive molecules like integrins [59]. Due to functions of CX3CL1 as chemo-attractant and adhesion molecule, it plays an important role in the process of recruiting cells from the blood stream to the site of action in an integrin-independent manner, e.g., the extravasation of CX3CR1-positive leukocytes [60]. CX3CL1 expression is mainly found in endothelial cells and neurons, but based on the involvement in inflammatory processes it is also expressed in tissue showing rather dense immune cell populations like the CNS, lung, cardiac muscles, liver, small bowel, colon, and pancreas [61].

## The role of CXCR3 and CX3CR1 chemokines in cancer

The role of the CXCR3 chemokine system in cancer biology may be a double-edged sword. On the one hand, the CXCR3 chemokines, especially CXCL9 and CXCL10, facilitate the recruitment of CXCR3-positive Th1, NK, and NKT cells as well as cytotoxic T lymphocytes to the tumor microenvironment, which trigger the development of a tumor-suppressive immune milieu [46, 62, 63]. On the other hand, tumor cells can exploit the CXCR3 receptor to escape from the primary tumor and to metastasize to niches with high CXCR3 ligand concentrations, e.g., to the lymph nodes or to the lungs [64–69]. Moreover, all three IFN-inducible CXCR3 ligands are anti-angiogenic *in vitro* and *in vivo*, which may result in undersupplied, stagnating tumors, but also in more aggressive tumor cells with metastasizing potential [70, 71].

Raising the intratumoral concentration of intact and functional CXCR3 chemokines, e.g., by inhibition of their proteolytic inactivation, might thus kill two birds with one stone: tumor-suppressive immune cells would be attracted to the tumor site, and CXCR3-positive tumor cells would be chemotactically prevented from escaping the primary cancer. This idea is supported both by preclinical and clinical findings. Overexpression of CXCR3 ligands in murine cancer models of ovarian, breast, skin, or colon cancer caused an enhanced Th1 and NK cell infiltration and less metastatic spread [72–75]. Moreover, in human cancers, overexpression of CXCL9 and CXCL10 is associated with a higher number of tumor-infiltrating lymphocytes and improved survival, e.g., in breast, ovarian, colon, lung, and several other cancers [67, 76–83]. Although CXCR3 expression by tumor cells is associated with worse prognosis in these cancers [67, 72, 84, 85] and CXCR3 ligands may also attract tumor-promoting regulatory T cells [86], the net effect of CXCR3 chemokine overexpression seems to favor tumor suppression. These results are confirmed by large-panel gene expression analyses of breast and ovarian cancer, in which CXCR3 chemokines represented the most upregulated genes in the tumors of those patients exhibiting the best prognosis [76, 87, 88].

However, CXCR3 chemokines do not only cause a tumor-suppressive milieu *per se*, they also contribute to the effect of multiple current cancer therapeutics. Immune checkpoint inhibitors of the PD-1/PD-L1 axis rely on immune cell attraction and on the intratumoral T cell activation by CXCR3 chemokines [20, 89–91]. Moreover, an increase in CXCR3 chemokine serum concentrations under therapy with checkpoint inhibitors is predictive for therapy response [92]. CDK4/6 inhibitors, which have emerged as a new cornerstone in the treatment of advanced estrogen receptor positive breast cancer, recruit cytotoxic T cells *via* induction of CXCR3 chemokines, which is indispensable for their therapeutic effect *in vivo* [93]. Inhibition of the poly[ADP-ribose] polymerase 1 (PARP1), which is now an established therapy in recurrent ovarian and metastatic BRCA-mutated breast cancer, also induces CXCR3 chemokines *via* the STING (stimulator of interferon genes) pathway in tumor cells, whereby the subsequent attraction of immune cells is critical for their function [94–99]. In all of these therapies, proteolytic inactivation of CXCR3 chemokines, thus, represents a new resistance mechanism, which renders inhibitors of CXCR3-chemokine cleaving proteases feasible adjuvants to all of these therapies.

CX3CL1 is also capable of recruiting tumor-suppressive immune cells that express the CX3CR1 receptor such as NK cells and cytotoxic T lymphocytes (CTLs) [21]. However, as described for the CXCR3 system, the CX3CR1 receptor is also able to facilitate tumor cell migration and, thereby, metastasis in CX3CL1-rich tissues, e.g. the bone or the brain [100, 101]. Moreover, expression of the transmembrane form of CX3CL1 in neurons, endothelial cells or peritoneal cells promotes tumor cell adhesion and site-specific metastasis of CX3CR1-expression prostate, ovarian, or pancreatic tumor cells [102–106].

Preclinical studies confirm the tumor-suppressive effect of CX3CL1 in several cancer models [107–112]. However, there are also studies in support of tumor-promoting effects of the CX3CL1-system: one study attributed a pro-metastatic function to the CX3CR1 receptor; however, the authors did not use an immuno-competent mouse model and, thereby, excluded the influence of immune modulatory effects [101]. Another study shows that CX3CL1 promotes the development of tumors, but not metastasis, in HER2 transgenic mice *via* transactivation of the EGF pathway [113]. This direct effect on tumor cells may relate to the fact that the tumor cells themselves express CX3CR1, whose activation directly triggers proliferation and migration [100, 101]. So far, it has not been satisfactorily clarified yet to what extent the two forms of CX3CL1 (membrane-bound *vs.* soluble) contribute to these different effects.

## Posttranslational modification of chemokines *via* proteolytic cleavage

### Cleavage of CXCR3 ligands

The activity of CXCR3 ligands is regulated within a complex and well-orchestrated network of different modulatory processes. Besides the regulation on both the transcriptional and translational level, modulation of chemokine activity by proteases comes more and more into focus. Moreover, a regulatory loop has been proposed in breast cancer, in which an increased expression of CXCL9 and/or CXCL10 leads to induction of cathepsin B gene expression and, in consequence cathepsin B protein levels, which then may reduce chemokine activity [84]. Similar effects were reported for the matrix metalloproteinases MMP-2 and MMP-9 in breast cancer, gastric cancer, colon cancer, and multiple myeloma [114–118]. Finally, a linear correlation of dipeptidyl peptidase 4 (DPP4) and CXCL10 expression was observed in ovarian cancer patients, suggesting a regulatory association between chemokine substrate and the protease DPP4 [33].

#### MMPs: N- and C-terminal cleavage of CXCR3 ligands

**Fig. 3:**
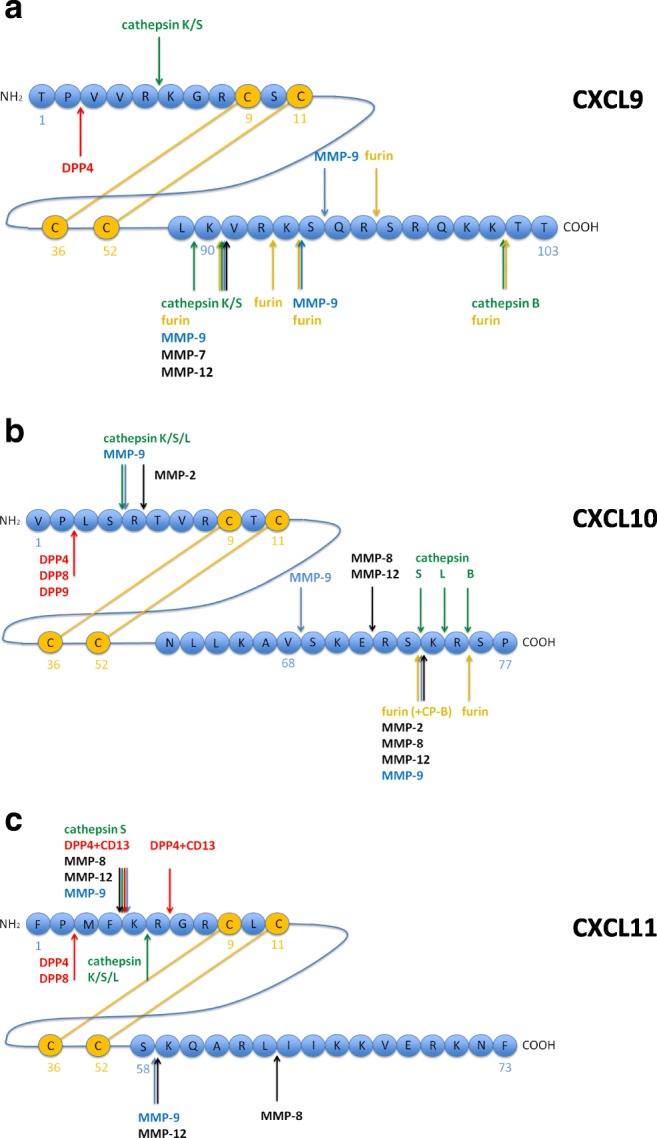
Reported cleavage sites in the CXC chemokines CXCL9, CXCL10, and CXCL11. N-terminal and C-terminal amino acids sequences are depicted for CXCL9 **A**, CXCL10 **B**, and CXCL11 **C**. Cleavage sites by DPPs and CD13 are indicated in red, by cathepsins in green, by furin (in the presence or absence of CP-B) in orange, by MMP-9 in blue and by other MMPs in black

Several members of the matrix metalloproteinase family carry out N- and C-terminal cleavages of CXCR3 ligands (Fig. 3). CXCL9 was reported to be C-terminally truncated by MMP-9 cleaving after Lys^90^, Lys^93^, and Ser^94^ [119]. In another study, in which several MMPs were analyzed, cleavage by MMP-7 and MMP-12 after Lys^90^ was observed as well. Interestingly, under the conditions used in the latter study, MMP-9 did not at all process CXCL9 [120].

For CXCL10, the cleavage sites for both MMP-8 and MMP-9 were mapped to Glu^71^↓Arg and Ser^73^↓Lys^74^. In addition, MMP-9 cleaved CXCL10 after Val^68^ [119]. In another study, MMP-9 was reported to cleave only at Ser^73^↓Lys^74^ coupled with an additional N-terminal cleavage after Ser^4^. N-terminal cleavage was observed also for MMP-2, after Arg^5^, together with C-terminal cleavage at Ser^73^↓Lys^74^ [121]. Finally, Cox and co-workers [120] reported that MMP-12 cleaves CXCL10 C-terminally after Glu^71^ and Ser^73^. Of note, in contrast to the study by van den Steen and co-workers [119], cleavage of CXCL10 by MMP-8 and MMP-9 was not observed, even with high enzyme-substrate ratios. MMP-7 degraded CXCL10 without generating stable intermediates.

For CXCL11, both N- and C-terminal processing by MMPs was reported. MMP-12, MMP-8, and MMP-9 cleave after Phe^4^ and after Ser^58^. Only MMP-8 displays an additional cleavage site at Leu^63^↓Ile^64^. Similar to CXCL10, MMP-7 mainly degrades CXCL11. However, a transient cleavage product could be detected by mass spectrometry corresponding to CXCL11_1–58_ [120]. N-terminally truncated forms, CXCL11_5–73_ and CXCL11_5–58_, have no detectable agonist activity in Ca^2+^ mobilization assays and only a very low activity in chemotactic migration assays [120] (Table 1).

**Table 1:** Characteristics of truncated chemokine forms

	Truncated form	Effects on chemokine-mediated processes	Reference
**CXCL9** (1–103)	6–90	Reduced induction of Ca^2+^ influx	[122]
	6–89	Reduced induction of Ca^2+^ influx	[122]
	3–103	Lack of chemotactic activity, reduced induction of Ca^2+^ influx, full angiostatic activity	[123]
	1-78^**a**^	Reduced induction of Ca^2+^ influx	[124]
**CXCL10** (1–77)	5–77	Lack of chemotactic activity, no induction of Ca^2+^ influx	[122]
	3–77	Reduced CXCR3 binding, reduced chemotactic activity, no induction of Ca^2+^ influx, full angiostatic activity, CXCR3 antagonist^**b**^	[123]
	1–73	Full chemotactic activity	[125]
**CXCL11** (1–73)	5–73	Reduced chemotactic activity, no induction of Ca^2+^ influx, CXCR3 antagonist^**c**^	[120]
	5–58	Lack of chemotactic activity, no induction of Ca^2+^ influx, no heparin binding	[120]
	3–73	Reduced CXCR3 binding, reduced chemotactic activity, no induction of Ca^2+^ influx, CXCR3 antagonist^**d**^	[123, 126]
	6–73	Lack of chemotactic activity	[122]
**mCX3CL1** (1–76)	5-78^**e**^	Lack of chemotactic activity, no induction of Ca^2+^ influx, CX3CR1 antagonist	[127]

^a^CXCL9_1–78_ corresponds to a truncated version of recombinantly expressed human CXCL9_1–103_ in Chinese hamster ovary cells generated by (an) unknown protease(s)

^b^CXCL10_3–77_ inhibits CXCL10_1–77_-mediated chemotaxis

^c^CXCL11_5–73_ inhibits CXCL11_1–73_-induced Ca^2+^ influx and CXCL11_1–73_-mediated chemotaxis

^d^CXCL11_3–73_ inhibits CXCL11_1–73_, but not CXCL10_1–77_-mediated chemotaxis

^e^The chemokine domain of CXCL11/fractalkine (aa 1–76 or its truncated form 5–76) was expressed as recombinant protein with a two amino acid extension at the C-terminus

#### Furin and carboxypeptidase: C-terminal truncation of CXCL10 does not change chemokine activity

In primary human keratinocytes, besides the full length form, a C-terminally truncated form of CXCL10, lacking the last four amino acids, was detected [128]. Whereas a broad range MMP inhibitor had no effect on processing, an inhibitor directed against furin completely inhibited processing. Subsequently, it was shown *in vitro* that furin is able to cleave off two amino acids, which generates the dibasic sequence Lys-Arg at the newly generated C-terminus representing a high-affinity substrate for carboxypeptidase B. This truncated CXCL10_1–73_ form retains its chemotactic activity [128] (Table 1). Under conditions, where full conversion of CXCL10_1–77_ to CXCL10_1–75_ was obtained, CXCL9 was only partially cleaved at five different positions. CXCL11 was no substrate for furin (and CB) (Fig. 3).

#### Cathepsins: cleavage modulates chemokine activity

Different internal cleavage sites for chemokines of the CXCR3 ligand family were mapped for some cysteine proteases of the cathepsin protease family [122] (Fig. 3). Interestingly, GAGs seem to stabilize cleavage intermediates or even prevent (further) processing in the C-terminal region of the cytokines. CXCL9 is internally cleaved by both cathepsin K and S near the N-terminus after Arg^5^ as well as in the C-terminal part after Leu^89^ and Lys^90^, respectively. Cathepsin B clips off the C-terminal two amino acids, whereas cathepsin L rapidly degrades CXCL9. Cathepsin K, L, and S cleave CXCL10 between Ser^4^ and Arg^5^. Furthermore, incubation of CXCL10 with cathepsin B, L, and S leads to C-terminally truncated forms lacking two, three and four amino acids, respectively. In CXCL11, major cleavage sites were mapped after Phe^4^ and Lys^5^ for cathepsin S. The latter cleavage site was also allocated to both cathepsin K and L. Some of the cathepsin-generated forms were further analyzed in cell biological assays in comparison with the respective chemotactically active full length cytokines: in Ca^2+^ mobilization assays, cathepsin S-cleaved CXCL9 (CXCL9_6–89_; CXCL9_6–90_) displayed considerably reduced chemokine activity, whereas cathepsin S-cleaved CXCL10 (CXCL_5–77_) showed no activity at all (Table 1). In migration assays, CXCL10_5–77_ (generated by incubation with either cathepsin S or L) and CXCL11_6–73_ (generated by cathepsin L), respectively, were inactive as well [128].

#### Dipeptidyl peptidases and aminopeptidase N (CD13): N-terminal cleavage impairs chemotactic function

Other naturally occurring cleavage products result from N-terminal cleavage events. In this respect, another protease family has gained attention in recent years, namely the exo-peptidase family of dipeptidyl peptidases (DPPs), which cleave dipeptides from the N-terminus. Especially, DPP4 removes N-terminal amino acids from all classical CXCR3 ligands (Fig. 3) causing impaired chemotactic activity as well as reduced Ca^2+^ influx [123, 126, 129]. Similarly, other members of the DPP family process CXCR3 chemokines, e.g., DPP8 inactivates CXCL10 and CXCL11 [130]. In addition, CXCL10 was suggested as a substrate for DPP9 [131]. Since the cleavage pattern is similar to that of DPP4 and DPP8, inactivation of the chemotactic ability of the cleavage product seems plausible as well. Aminopeptidase N (CD13), which removes single N-terminal residues, generates a truncated version of CXCL11 devoid of any chemotactic activity or binding to the CXCR3 receptor [132] (Table 1).

### Cleavage of CX3CL1

**Fig. 4:**
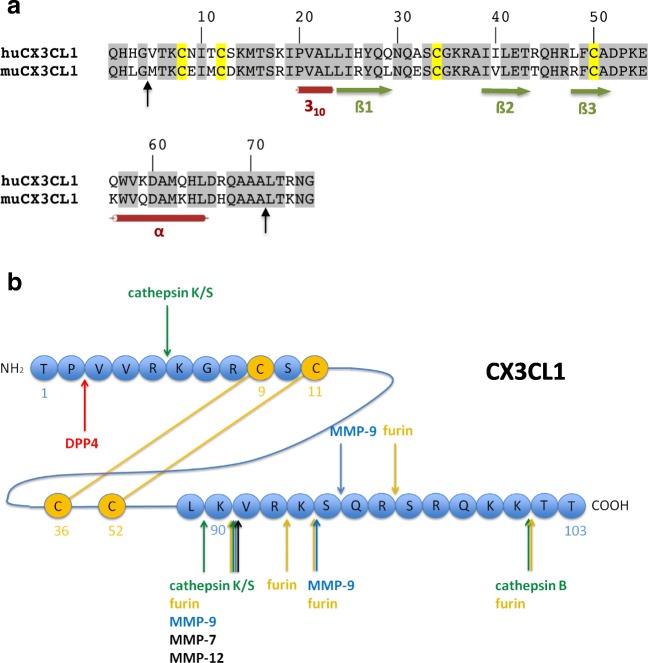
Cleavage of CX3CL1/fractalkine by proteases. **A** Alignment of the cytokine domain of human and murine CX3CL1. Secondary structure elements are indicated by green arrows (ß-strands) and red cylinders (α-helix and 3_10_-helix). The cleavage sites of MMP-2 (black arrows) were determined *via* cleavage of the murine recombinant ectodomain of CX3CL1 with human MMP-2. **B** Schematic representation of CX3CL1. The N-terminally located cytokine domain (aa 1–76) is linked to the transmembrane domain (plus short cytoplasmic tail) *via* a mucin-like stalk. N-terminal and C-terminal amino acid sequences of the cytokine domain of murine CX3CL1 are depicted. The cytokine domain can be shedded either together with the mucin-like domains by cathepsin S (green) as well as ADAM10 and ADAM17 (violet) or as isolated domain by MMP-2 (black; Ala^71^↓Leu^72^). The proteolytic cleavage by MMP-2 near the N-terminus (black; Gly^4^↓et^5^ results in inactivation of the cytokine

For CX3CL1, proteolytic cleavages play a major role in revealing the full potential function of the protein. Proteolytic cleavages convert the membrane-bound form to the soluble form, especially the metalloproteases ADAM17 and ADAM10 [133–135]. Activity of these ADAMs releases a soluble form of CX3CL1 encompassing the chemokine domain and large parts of the mucin-like stalk (Fig. 4). Since addition of ADAM10 and/or ADAM17 inhibitors did not completely prevent the release of soluble CX3CL1, other proteases were proposed to be involved in the shedding process as well [136]. In an unbiased mass spectrometry-based substrate screen for MMP-2, CX3CL1 was identified as a substrate for this matrix metalloproteinase. Cleavage by MMP-2 results in the release of a soluble form of CX3CL1 lacking the mucin-like stalk [57]. The MMP-mediated release of CX3CL1 peptides was validated *in vitro* by the addition of TIMP2 and TIMP3, natural inhibitors of MMP-2 and other MMPs [137]. MMP-2 also generates an N-terminally truncated form of CX3CL1 lacking four amino acids [57]. Recombinantly expressed CX3CL1_5–78_ did neither induce Ca^2+^ influx nor displayed any chemotactic activity. Furthermore, competitive chemotaxis assays revealed CX3CL1_5–78_ as a CX3CR1 antagonist [127] (Table 1).

Another sheddase for CX3CL1 was found in the context of microglia signaling, where cathepsin S was shown to be a major regulator of generation of soluble CX3CL1 [138]. CX3CL1 may also be targeted by MMP-9: in a non-obese diabetic (NOD) mouse model, Wildenberg and co-workers [139] detected 17 kDa and 19 kDa cleavage products of CX3CL1 in the salivary gland, which was accompanied by an increased gelatinase and α-secretase activity. *In vitro* cleavage assays excluded ADAM10 and 17 as well as MMP-2 as responsible enzymes and, thus, pointed to MMP-9 as one of the responsible proteases for this organ-specific cleavage *in vivo*. It should be noted, however, that *in vitro* MMP-9 led to the degradation of the target protein and its inactivation [139].

All these data suggest that proteases have a strong impact on CX3CL1 release suggesting involvement in the modulation of the immune response. Nevertheless, until now it is not fully understood, whether the membrane-bound or soluble form of CX3CL1 has a more regulatory impact.

### *In vivo* relevance of proteolytic chemokine processing

While cleavage and its functional consequences of CXCR3 and CX3CR1 chemokines by proteases have been rather well characterized on the biochemical level, there is hardly any *in vivo* data on the impact of these cleavage processes on tumor-immune interactions.

Only indirect data exists showing that protease expression is associated with unfavorable prognosis and less lymphocytic tumor infiltrates in certain cancers. Regarding matrix metalloproteinases, a recent study showed that inhibition of MMP-9 by a monoclonal antibody in an immuno-competent model of HER2-positive breast cancer increased tumor-suppressive T cell infiltration and CXCR3 chemokine expression. Whether MMP-9-antagonism increases T cell trafficking due to CXCR3 chemokine cleavage *in vivo* was, however, not demonstrated [140].

Most detailed *in vivo* data are available for the DPP4-mediated cleavage of CXCR3 chemokines. In syngeneic models of melanoma and colorectal cancer (B16F10 and CT26 models, respectively), inhibition of DPP4 by sitagliptin or DPP4 knockout led to enhanced T cell infiltration, impaired tumor growth and less metastatic spread [19]. This effect was abrogated in Cxcr3^−/−^ mice and dependent on N-terminal CXCL10 truncation. Moreover, immune checkpoint inhibitor therapy was improved by DPP4 inhibition with sitagliptin [19]. These data were confirmed in both a xenograft and a fully immuno-competent model of hepatocellular carcinoma, in which DPP4 inhibition also impaired tumor growth by enhancing the CXCR3-mediated NK and T cell infiltration [141]. However, in another HCC model as well as in a triple-negative breast cancer model, the tumor-suppressive effect of sitagliptin could not be traced back to the CXCR3 chemokine cleavage, but was instead dependent on a CCL11-mediated higher eosinophilic infiltration [142]. Seemingly, the underlying mechanisms of DPP4-mediated anti-tumor immune modulation could be different across tumor types (and maybe across mouse models). In human ovarian cancer, the existence of DPP4-mediated CXCL10 cleavage products was demonstrated, suggesting a relevant role for these mechanisms also in patients [143].

## Conclusion

Taken together, CXCR3 and CX3CR1 chemokines are part of a complex regulatory network which orchestrates a broad variety of physiologic functions that can modulate the anti-tumoral immune response. On the one hand, chemokines help to shape the tumor-microenvironment by regulating the amount and type of infiltrating lymphocytes. On the other hand, they are enhancing cell biological processes such as proliferation, invasion, and angiogenesis and, thereby, promote tumor aggressiveness and metastatic potential. Posttranslational modifications of these chemokines by tumor cells, such as proteolytic cleavage, can exert a strong regulatory impact resulting in a shift towards a tumor-promoting environment. Proteolytic cleavage of the tumor-suppressive CXCR3 and CX3CR1 chemokines impairs their functions and, on top of this, in feedback loops, the chemokines may even lead to increased expression of the proteases targeting themselves. Thus, we suggest that cleavage of the ligands of the CXCR3 and CX3CR1 chemokine systems represents a potent immune escape mechanism in cancer. Deeper knowledge of the mechanisms behind this also provides a panel of interesting novel target structures to support existing therapies or develop new ones.

## References

[1] Tyzzer EE (1916). Tumor immunity. American Journal of Cancer Research.

[2] Decker WK, da Silva RF, Sanabria MH, Angelo LS, Guimaraes F, Burt BM (2017). Cancer immunotherapy: historical perspective of a clinical revolution and emerging preclinical animal models. Frontiers in Immunology.

[3] Zhang L, Conejo-Garcia JR, Katsaros D, Gimotty PA, Massobrio M, Regnani G, Makrigiannakis A, Gray H, Schlienger K, Liebman MN, Rubin SC, Coukos G (2003). Intratumoral T cells, recurrence, and survival in epithelial ovarian cancer. New England Journal of Medicine.

[4] Dieci MV, Radosevic-Robin N, Fineberg S, van den Eynden G, Ternes N, Penault-Llorca F (2018). Update on tumor-infiltrating lymphocytes (TILs) in breast cancer, including recommendations to assess TILs in residual disease after neoadjuvant therapy and in carcinoma in situ: a report of the international immuno-oncology biomarker working group on Bre. Seminars in Cancer Biology.

[5] Pages F, Mlecnik B, Marliot F, Bindea G, Ou FS, Bifulco C (2018). International validation of the consensus immunoscore for the classification of colon cancer: a prognostic and accuracy study. Lancet.

[6] Fridman WH, Pages F, Sautes-Fridman C, Galon J (2012). The immune contexture in human tumours: impact on clinical outcome. Nature Reviews. Cancer.

[7] Fridman WH, Zitvogel L, Sautes-Fridman C, Kroemer G (2017). The immune contexture in cancer prognosis and treatment. Nature Reviews. Clinical Oncology.

[8] Velcheti V, Schalper K (2016). Basic overview of current immunotherapy approaches in cancer. American Society of Clinical Oncology Educational Book.

[9] Loibl S, Gianni L (2017). HER2-positive breast cancer. The Lancet.

[10] Postow MA, Callahan MK, Wolchok JD (2015). Immune checkpoint blockade in cancer therapy. Journal of Clinical Oncology : Official Journal of the American Society of Clinical Oncology.

[11] Galluzzi L, Zitvogel L, Kroemer G (2016). Immunological mechanisms underneath the efficacy of cancer therapy. Cancer Immunology Research.

[12] Abastado J-P (2012). The next challenge in cancer immunotherapy: controlling T-cell traffic to the tumor. Cancer Research.

[13] Melero I, Rouzaut A, Motz GT, Coukos G (2014). T-cell and NK-cell infiltration into solid tumors: a key limiting factor for efficacious cancer immunotherapy. Cancer Discovery.

[14] de Melo Gagliato D, Cortes J, Curigliano G, Loi S, Denkert C, Perez-Garcia J, Holgado E (2017). Tumor-infiltrating lymphocytes in breast cancer and implications for clinical practice. Biochimica Et Biophysica Acta. Reviews on Cancer.

[15] Galon J, Bruni D (2019). Approaches to treat immune hot, altered and cold tumours with combination immunotherapies. Nature Reviews. Drug Discovery.

[16] Zlotnik A, Yoshie O (2012). The chemokine superfamily revisited. Immunity.

[17] Nagarsheth N, Wicha MS, Zou W (2017). Chemokines in the cancer microenvironment and their relevance in cancer immunotherapy. Nature Reviews Immunology.

[18] Wolf M, Albrecht S, Märki C (2008). Proteolytic processing of chemokines: implications in physiological and pathological conditions. The International Journal of Biochemistry & Cell Biology.

[19] Barreira da Silva Rosa, Laird Melissa E, Yatim Nader, Fiette Laurence, Ingersoll Molly A, Albert Matthew L (2015). Dipeptidylpeptidase 4 inhibition enhances lymphocyte trafficking, improving both naturally occurring tumor immunity and immunotherapy. Nature Immunology.

[20] Chow MT, Ozga AJ, Servis RL, Frederick DT, Lo JA, Fisher DE, Freeman GJ, Boland GM, Luster AD (2019). Intratumoral activity of the CXCR3 chemokine system is required for the efficacy of anti-PD-1 therapy. Immunity.

[21] Chen DS, Mellman I (2013). Oncology meets immunology: the cancer-immunity cycle. Immunity.

[22] Metzemaekers M, Vanheule V, Janssens R, Struyf S, Proost P (2017). Overview of the mechanisms that may contribute to the non-redundant activities of interferon-inducible CXC chemokine receptor 3 ligands. Frontiers in Immunology.

[23] Palomino DCT, Marti LC (2015). Chemokines and immunity. Einstein (São Paulo).

[24] Poeta VM, Massara M, Capucetti A, Bonecchi R (2019). Chemokines and chemokine receptors: new targets for cancer immunotherapy. Frontiers in Immunology.

[25] Moser B, Willimann K (2004). Chemokines: role in inflammation and immune surveillance. Annals of the Rheumatic Diseases.

[26] Turner MD, Nedjai B, Hurst T, Pennington DJ (2014). Cytokines and chemokines: At the crossroads of cell signalling and inflammatory disease. Biochimica et Biophysica Acta, Molecular Cell Research.

[27] Esche C, Stellato C, Beck LA (2005). Chemokines: key players in innate and adaptive immunity. The Journal of Investigative Dermatology.

[28] Griffith JW, Sokol CL, Luster AD (2014). Chemokines and chemokine receptors: positioning cells for host defense and immunity. Annual Review of Immunology.

[29] Dimberg A (2010). Chemokines in angiogenesis. Current Topics in Microbiology and Immunology.

[30] Mehrad B, Keane MP, Strieter RM (2007). Chemokines as mediators of angiogenesis. Thrombosis and Haemostasis.

[31] Eberlein J, Nguyen TT, Victorino F, Golden-Mason L, Rosen HR, Homann D (2010). Comprehensive assessment of chemokine expression profiles by flow cytometry. Journal of Clinical Investigation.

[32] Zlotnik A, Yoshie O, Nomiyama H (2006). The chemokine and chemokine receptor superfamilies and their molecular evolution. Genome Biology.

[33] Mortier A, Van Damme J, Proost P (2012). Overview of the mechanisms regulating chemokine activity and availability. Immunology Letters.

[34] Nibbs RJB, Graham GJ (2013). Immune regulation by atypical chemokine receptors. Nature Reviews Immunology.

[35] Hughes CE, Nibbs RJB (2018). A guide to chemokines and their receptors. FEBS Journal.

[36] Legler DF, Thelen M (2018). New insights in chemokine signaling. F1000Research.

[37] Steen A, Larsen O, Thiele S, Rosenkilde MM (2014). Biased and G protein-independent signaling of chemokine receptors. Frontiers in Immunology.

[38] Miller MC, Mayo KH (2017). Chemokines from a structural perspective. International Journal of Molecular Sciences.

[39] Zlotnik A, Yoshie O (2000). Chemokines: a new classification system and their role in immunity. Immunity.

[40] Rajagopalan L, Rajarathnam K (2006). Structural basis of chemokine receptor function–a model for binding affinity and ligand selectivity. Bioscience Reports.

[41] Sanchez J, Huma e Z, Lane JR, Liu X, Bridgford JL, Payne RJ (2019). Evaluation and extension of the two-site, two-step model for binding and activation of the chemokine receptor CCR1. The Journal of Biological Chemistry.

[42] Proudfoot AEI, Johnson Z, Bonvin P, Handel TM (2017). Glycosaminoglycan interactions with chemokines add complexity to a complex system. Pharmaceuticals.

[43] Strieter RM, Polverini PJ, Kunkel SL, Arenberg DA, Burdick MD, Kasper J, Dzuiba J, van Damme J, Walz A, Marriott D, Chan SY, Roczniak S, Shanafelt AB (1995). The functional role of the ELR motif in CXC chemokine-mediated angiogenesis. Journal of Biological Chemistry.

[44] Billottet C, Quemener C, Bikfalvi A (2013). CXCR3, a double-edged sword in tumor progression and angiogenesis. Biochimica et Biophysica Acta.

[45] Ehlert JE, Addison CA, Burdick MD, Kunkel SL, Strieter RM (2004). Identification and partial characterization of a variant of human CXCR3 generated by posttranscriptional exon skipping. The Journal of Immunology.

[46] Wendel M, Galani IE, Suri-Payer E, Cerwenka A (2008). Natural killer cell accumulation in tumors is dependent on IFN-gamma and CXCR3 ligands. Cancer Research.

[47] Groom JR, Luster AD (2011). CXCR3 ligands: redundant, collaborative and antagonistic functions. Immunology and Cell Biology.

[48] Cox MA, Jenh CH, Gonsiorek W, Fine J, Narula SK, Zavodny PJ, Hipkin RW (2001). Human interferon-inducible 10-kDa protein and human interferon-inducible T cell alpha chemoattractant are allotopic ligands for human CXCR3: differential binding to receptor states. Molecular Pharmacology.

[49] Colvin RA, Campanella GSV, Sun J, Luster AD (2004). Intracellular domains of CXCR3 that mediate CXCL9, CXCL10, and CXCL11 function. Journal of Biological Chemistry.

[50] Zohar Y, Wildbaum G, Novak R, Salzman AL, Thelen M, Alon R, Barsheshet Y, Karp CL, Karin N (2014). CXCL11-dependent induction of FOXP3-negative regulatory T cells suppresses autoimmune encephalomyelitis. Journal of Clinical Investigation.

[51] Groom JR, Richmond J, Murooka TT, Sorensen EW, Sung JH, Bankert K, von Andrian UH, Moon JJ, Mempel TR, Luster AD (2012). CXCR3 chemokine receptor-ligand interactions in the lymph node optimize CD4+ T helper 1 cell differentiation. Immunity.

[52] Groom JR, Luster AD (2012). CXCR3 in T cell function. Experimental Cell Research.

[53] Wojdasiewicz P, Poniatowski ŁA, Kotela A, Deszczyński J, Kotela I, Szukiewicz D (2014). The chemokine CX3CL1 (fractalkine) and its receptor CX3CR1: occurrence and potential role in osteoarthritis. Archivum Immunologiae et Therapiae Experimentalis.

[54] Bazan JF, Bacon KB, Hardiman G, Wang W, Soo K, Rossi D, Greaves DR, Zlotnik A, Schall TJ (1997). A new class of membrane-bound chemokine with a CX3C motif. Nature.

[55] Kim K-W, Vallon-Eberhard A, Zigmond E, Farache J, Shezen E, Shakhar G, Ludwig A, Lira SA, Jung S (2011). In vivo structure/function and expression analysis of the CX3C chemokine fractalkine. Blood.

[56] Imai T, Hieshima K, Haskell C, Baba M, Nagira M, Nishimura M, Kakizaki M, Takagi S, Nomiyama H, Schall TJ, Yoshie O (1997). Identification and molecular characterization of fractalkine receptor CX3CR1, which mediates both leukocyte migration and adhesion. Cell.

[57] Dean RA, Overall CM (2007). Proteomics discovery of metalloproteinase substrates in the cellular context by iTRAQ labeling reveals a diverse MMP-2 substrate degradome. Molecular & Cellular Proteomics : MCP.

[58] Ostuni MA, Guellec J, Hermand P, Durand P, Combadiere C, Pincet F, Deterre P (2014). CX3CL1, a chemokine finely tuned to adhesion: critical roles of the stalk glycosylation and the membrane domain. Biology Open.

[59] Fujita M, Takada YK, Takada Y (2014). The chemokine fractalkine can activate integrins without CX3CR1 through direct binding to a ligand-binding site distinct from the classical RGD-binding site. PLoS One.

[60] Haskell CA, Cleary MD, Charo IF (2000). Unique role of the chemokine domain of fractalkine in cell capture. Kinetics of receptor dissociation correlate with cell adhesion. The Journal of Biological Chemistry.

[61] Poniatowski ŁA, Wojdasiewicz P, Krawczyk M, Szukiewicz D, Gasik R, Kubaszewski Ł, Kurkowska-Jastrzębska I (2017). Analysis of the role of CX3CL1 (Fractalkine) and its receptor CX3CR1 in traumatic brain and spinal cord injury: Insight into recent advances in actions of neurochemokine agents. Molecular Neurobiology.

[62] Chheda ZS, Sharma RK, Jala VR, Luster AD, Haribabu B (2016). Chemoattractant receptors BLT1 and CXCR3 regulate antitumor immunity by facilitating CD8+ T cell migration into tumors. Journal of Immunology (Baltimore, Md. : 1950).

[63] Tokunaga R, Zhang W, Naseem M, Puccini A, Berger MD, Soni S, McSkane M, Baba H, Lenz HJ (2018). CXCL9, CXCL10, CXCL11/CXCR3 axis for immune activation – a target for novel cancer therapy. Cancer Treatment Reviews.

[64] Walser TC, Rifat S, Ma X, Kundu N, Ward C, Goloubeva O, Johnson MG, Medina JC, Collins TL, Fulton AM (2006). Antagonism of CXCR3 inhibits lung metastasis in a murine model of metastatic breast cancer. Cancer Research.

[65] Kawada K, Hosogi H, Sonoshita M, Sakashita H, Manabe T, Shimahara Y, Sakai Y, Takabayashi A, Oshima M, Taketo MM (2007). Chemokine receptor CXCR3 promotes colon cancer metastasis to lymph nodes. Oncogene.

[66] Cambien B, Karimdjee BF, Richard-Fiardo P, Bziouech H, Barthel R, Millet MA, Martini V, Birnbaum D, Scoazec JY, Abello J, Saati TA, Johnson MG, Sullivan TJ, Medina JC, Collins TL, Schmid-Alliana A, Schmid-Antomarchi H (2009). Organ-specific inhibition of metastatic colon carcinoma by CXCR3 antagonism. British Journal of Cancer.

[67] Ma X, Norsworthy K, Kundu N, Rodgers WH, Gimotty PA, Goloubeva O, Lipsky M, Li Y, Holt D, Fulton A (2009). CXCR3 expression is associated with poor survival in breast cancer and promotes metastasis in a murine model. Molecular Cancer Therapeutics.

[68] Pradelli E, Karimdjee-Soilihi B, Michiels JF, Ricci JE, Millet MA, Vandenbos F, Sullivan TJ, Collins TL, Johnson MG, Medina JC, Kleinerman ES, Schmid-Alliana A, Schmid-Antomarchi H (2009). Antagonism of chemokine receptor CXCR3 inhibits osteosarcoma metastasis to lungs. International Journal of Cancer.

[69] Kawada K, Taketo MM (2011). Significance and mechanism of lymph node metastasis in cancer progression. Cancer Research.

[70] Arenberg D, White E, Burdick M, Strom S, Strieter R (2001). Improved survival in tumor-bearing SCID mice treated with interferon-γ-inducible protein 10 (IP-10/CXCL10). Cancer Immunology, Immunotherapy.

[71] Pan J, Burdick MD, Belperio JA, Xue YY, Gerard C, Sharma S (2006). CXCR3/CXCR3 ligand biological axis impairs RENCA tumor growth by a mechanism of immunoangiostasis. Journal of Immunology (Baltimore, Md. : 1950).

[72] Yang X, Chu Y, Wang Y, Zhang R, Xiong S (2006). Targeted in vivo expression of IFN-gamma-inducible protein 10 induces specific antitumor activity. Journal of Leukocyte Biology.

[73] Gorbachev AV, Kobayashi H, Kudo D, Tannenbaum CS, Finke JH, Shu S (2007). CXC chemokine ligand 9/monokine induced by IFN- production by tumor cells is critical for T cell-mediated suppression of cutaneous tumors. The Journal of Immunology.

[74] Zumwalt TJ, Arnold M, Goel A, Boland CR (2015). Active secretion of CXCL10 and CCL5 from colorectal cancer microenvironments associates with granzyme B+ CD8+ T-cell infiltration. Oncotarget.

[75] Au KK, Peterson N, Truesdell P, Reid-Schachter G, Khalaj K, Ren R (2017). CXCL10 alters the tumour immune microenvironment and disease progression in a syngeneic murine model of high-grade serous ovarian cancer. Gynecologic Oncology.

[76] Specht K, Harbeck N, Smida J, Annecke K, Reich U, Naehrig J, Langer R, Mages J, Busch R, Kruse E, Klein-Hitpass L, Schmitt M, Kiechle M, Hoefler H (2009). Expression profiling identifies genes that predict recurrence of breast cancer after adjuvant CMF-based chemotherapy. Breast Cancer Research and Treatment.

[77] Denkert C, Loibl S, Noske A, Roller M, Müller BM, Komor M, Budczies J, Darb-Esfahani S, Kronenwett R, Hanusch C, von Törne C, Weichert W, Engels K, Solbach C, Schrader I, Dietel M, von Minckwitz G (2010). Tumor-associated lymphocytes as an independent predictor of response to neoadjuvant chemotherapy in breast cancer. Journal of Clinical Oncology: Official Journal of the American Society of Clinical Oncology.

[78] Mlecnik B, Tosolini M, Charoentong P, Kirilovsky A, Bindea G, Berger A, Camus M, Gillard M, Bruneval P, Fridman W–H, Pagès F, Trajanoski Z, Galon J (2010). Biomolecular network reconstruction identifies T-cell homing factors associated with survival in colorectal cancer. Gastroenterology.

[79] Denkert C, von Minckwitz G, Brase JC, Sinn BV, Gade S, Kronenwett R (2015). Tumor-infiltrating lymphocytes and response to neoadjuvant chemotherapy with or without carboplatin in human epidermal growth factor receptor 2-positive and triple-negative primary breast cancers. Journal of Clinical Oncology: Official Journal of the American Society of Clinical Oncology.

[80] Bronger H, Singer J, Windmüller C, Reuning U, Zech D, Delbridge C, Dorn J, Kiechle M, Schmalfeldt B, Schmitt M, Avril S (2016). CXCL9 and CXCL10 predict survival and are regulated by cyclooxygenase inhibition in advanced serous ovarian cancer. British Journal of Cancer.

[81] Sato Y, Motoyama S, Nanjo H, Wakita A, Yoshino K, Sasaki T, Nagaki Y, Liu J, Imai K, Saito H, Minamiya Y (2016). CXCL10 expression status is prognostic in patients with advanced thoracic esophageal squamous cell carcinoma. Annals of Surgical Oncology.

[82] Wu Z, Huang X, Han X, Li Z, Zhu Q, Yan J, Yu S, Jin Z, Wang Z, Zheng Q, Wang Y (2016). The chemokine CXCL9 expression is associated with better prognosis for colorectal carcinoma patients. Biomedicine & Pharmacotherapy.

[83] Cao Y, Huang H, Wang Z, Zhang G (2017). The inflammatory CXC chemokines, GROalpha(high), IP-10(low), and MIG(low), in tumor microenvironment can be used as new indicators for non-small cell lung cancer progression. Immunological Investigations.

[84] Bronger H, Karge A, Dreyer T, Zech D, Kraeft S, Avril S, Kiechle M, Schmitt M (2017). Induction of cathepsin B by the CXCR3 chemokines CXCL9 and CXCL10 in human breast cancer cells. Oncology Letters.

[85] Windmüller C, Zech D, Avril S, Boxberg M, Dawidek T, Schmalfeldt B, Schmitt M, Kiechle M, Bronger H (2017). CXCR3 mediates ascites-directed tumor cell migration and predicts poor outcome in ovarian cancer patients. Oncogenesis.

[86] Redjimi N, Raffin C, Raimbaud I, Pignon P, Matsuzaki J, Odunsi K, Valmori D, Ayyoub M (2012). CXCR3+ T regulatory cells selectively accumulate in human ovarian carcinomas to limit type I immunity. Cancer Research.

[87] Wang C, Armasu SM, Kalli KR, Maurer MJ, Heinzen EP, Keeney GL, Cliby WA, Oberg AL, Kaufmann SH, Goode EL (2017). Pooled clustering of high-grade serous ovarian cancer gene expression leads to novel consensus subtypes associated with survival and surgical outcomes. Clinical Cancer Research.

[88] Zhang AW, McPherson A, Milne K, Kroeger DR, Hamilton PT, Miranda A, Funnell T, Little N, de Souza CPE, Laan S, LeDoux S, Cochrane DR, Lim JLP, Yang W, Roth A, Smith MA, Ho J, Tse K, Zeng T, Shlafman I, Mayo MR, Moore R, Failmezger H, Heindl A, Wang YK, Bashashati A, Grewal DS, Brown SD, Lai D, Wan ANC, Nielsen CB, Huebner C, Tessier-Cloutier B, Anglesio MS, Bouchard-Côté A, Yuan Y, Wasserman WW, Gilks CB, Karnezis AN, Aparicio S, McAlpine JN, Huntsman DG, Holt RA, Nelson BH, Shah SP (2018). Interfaces of malignant and immunologic clonal dynamics in ovarian cancer. Cell.

[89] Peng W, Liu C, Xu C, Lou Y, Chen J, Yang Y, Yagita H, Overwijk WW, Lizee G, Radvanyi L, Hwu P (2012). PD-1 blockade enhances T-cell migration to tumors by elevating IFN-gamma inducible chemokines. Cancer Research.

[90] Peng D, Kryczek I, Nagarsheth N, Zhao L, Wei S, Wang W, Sun Y, Zhao E, Vatan L, Szeliga W, Kotarski J, Tarkowski R, Dou Y, Cho K, Hensley-Alford S, Munkarah A, Liu R, Zou W (2015). Epigenetic silencing of TH1-type chemokines shapes tumour immunity and immunotherapy. Nature.

[91] Seo H, Kim BS, Bae EA, Min BS, Han YD, Shin SJ, Kang CY (2018). IL21 therapy combined with PD-1 and Tim-3 blockade provides enhanced NK cell antitumor activity against MHC class I-deficient tumors. Cancer Immunology Research.

[92] Oyanagi J, Koh Y, Sato K, Mori K, Teraoka S, Akamatsu H, Kanai K, Hayata A, Tokudome N, Akamatsu K, Nakanishi M, Ueda H, Yamamoto N (2019). Predictive value of serum protein levels in patients with advanced non-small cell lung cancer treated with nivolumab. Lung Cancer.

[93] Goel S, DeCristo MJ, Watt AC, BrinJones H, Sceneay J, Li BB, Khan N, Ubellacker JM, Xie S, Metzger-Filho O, Hoog J, Ellis MJ, Ma CX, Ramm S, Krop IE, Winer EP, Roberts TM, Kim HJ, McAllister SS, Zhao JJ (2017). CDK4/6 inhibition triggers anti-tumour immunity. Nature.

[94] Ding L, Kim HJ, Wang Q, Kearns M, Jiang T, Ohlson CE, Li BB, Xie S, Liu JF, Stover EH, Howitt BE, Bronson RT, Lazo S, Roberts TM, Freeman GJ, Konstantinopoulos PA, Matulonis UA, Zhao JJ (2018). PARP inhibition elicits STING-dependent antitumor immunity in Brca1-deficient ovarian cancer. Cell Reports.

[95] Chabanon RM, Muirhead G, Krastev DB, Adam J, Morel D, Garrido M, Lamb A, Hénon C, Dorvault N, Rouanne M, Marlow R, Bajrami I, Cardeñosa ML, Konde A, Besse B, Ashworth A, Pettitt SJ, Haider S, Marabelle A, Tutt ANJ, Soria JC, Lord CJ, Postel-Vinay S (2019). PARP inhibition enhances tumor cell-intrinsic immunity in ERCC1-deficient non-small cell lung cancer. The Journal of Clinical Investigation.

[96] Pantelidou C, Sonzogni O, De Oliveria Taveira M, Mehta AK, Kothari A, Wang D (2019). PARP inhibitor efficacy depends on CD8(+) T-cell recruitment via intratumoral STING pathway activation in BRCA-deficient models of triple-negative breast cancer. Cancer Discovery.

[97] Sen T, Rodriguez BL, Chen L, Corte CMD, Morikawa N, Fujimoto J, Cristea S, Nguyen T, Diao L, Li L, Fan Y, Yang Y, Wang J, Glisson BS, Wistuba II, Sage J, Heymach JV, Gibbons DL, Byers LA (2019). Targeting DNA damage response promotes antitumor immunity through STING-mediated T-cell activation in small cell lung cancer. Cancer Discovery.

[98] Shen J, Zhao W, Ju Z, Wang L, Peng Y, Labrie M, Yap TA, Mills GB, Peng G (2019). PARPi triggers the STING-dependent immune response and enhances the therapeutic efficacy of immune checkpoint blockade independent of BRCAness. Cancer Research.

[99] Wang Z, Sun K, Xiao Y, Feng B, Mikule K, Ma X, Feng N, Vellano CP, Federico L, Marszalek JR, Mills GB, Hanke J, Ramaswamy S, Wang J (2019). Niraparib activates interferon signaling and potentiates anti-PD-1 antibody efficacy in tumor models. Scientific Reports.

[100] Andre F, Cabioglu N, Assi H, Sabourin JC, Delaloge S, Sahin A, Broglio K, Spano JP, Combadiere C, Bucana C, Soria JC, Cristofanilli M (2006). Expression of chemokine receptors predicts the site of metastatic relapse in patients with axillary node positive primary breast cancer. Annals of Oncology.

[101] Jamieson-Gladney WL, Zhang Y, Fong AM, Meucci O, Fatatis A (2011). The chemokine receptor CX(3)CR1 is directly involved in the arrest of breast cancer cells to the skeleton. Breast Cancer Research.

[102] Shulby SA, Dolloff NG, Stearns ME, Meucci O, Fatatis A (2004). CX3CR1-fractalkine expression regulates cellular mechanisms involved in adhesion, migration, and survival of human prostate cancer cells. Cancer Research.

[103] Marchesi F, Piemonti L, Fedele G, Destro A, Roncalli M, Albarello L, Doglioni C, Anselmo A, Doni A, Bianchi P, Laghi L, Malesci A, Cervo L, Malosio ML, Reni M, Zerbi A, di Carlo V, Mantovani A, Allavena P (2008). The chemokine receptor CX3CR1 is involved in the neural tropism and malignant behavior of pancreatic ductal adenocarcinoma. Cancer Research.

[104] Marchesi F, Piemonti L, Mantovani A, Allavena P (2010). Molecular mechanisms of perineural invasion, a forgotten pathway of dissemination and metastasis. Cytokine & Growth Factor Reviews.

[105] Kim M, Rooper L, Xie J, Kajdacsy-Balla AA, Barbolina MV (2012). Fractalkine receptor CX(3)CR1 is expressed in epithelial ovarian carcinoma cells and required for motility and adhesion to peritoneal mesothelial cells. Molecular Cancer Research.

[106] Gurler Main H, Xie J, Muralidhar GG, Elfituri O, Xu H, Kajdacsy-Balla AA, Barbolina MV (2017). Emergent role of the fractalkine axis in dissemination of peritoneal metastasis from epithelial ovarian carcinoma. Oncogene.

[107] Kanagawa N, Niwa M, Hatanaka Y, Tani Y, Nakagawa S, Fujita T, Yamamoto A, Okada N (2007). CC-chemokine ligand 17 gene therapy induces tumor regression through augmentation of tumor-infiltrating immune cells in a murine model of preexisting CT26 colon carcinoma. International Journal of Cancer.

[108] Ren T, Chen Q, Tian Z, Wei H (2007). Down-regulation of surface fractalkine by RNA interference in B16 melanoma reduced tumor growth in mice. Biochemical and Biophysical Research Communications.

[109] Tang L, Hu H, Hu P, Lan Y, Peng M, Chen M (2007). Gene therapy with CX3CL1/fractalkine induces antitumor immunity to regress effectively mouse hepatocellular carcinoma. Gene Therapy.

[110] Vitale S, Cambien B, Karimdjee BF, Barthel R, Staccini P, Luci C, Breittmayer V, Anjuere F, Schmid-Alliana A, Schmid-Antomarchi H (2007). Tissue-specific differential antitumour effect of molecular forms of fractalkine in a mouse model of metastatic colon cancer. Gut.

[111] Yu YRA, Fong AM, Combadiere C, Gao JL, Murphy PM, Patel DD (2007). Defective antitumor responses in CX3CR1-deficient mice. International Journal of Cancer.

[112] Richard-Fiardo P, Cambien B, Pradelli E, Beilvert F, Pitard B, Schmid-Antomarchi H, Schmid-Alliana A (2011). Effect of fractalkine-Fc delivery in experimental lung metastasis using DNA/704 nanospheres. Cancer Gene Therapy.

[113] Tardáguila M, Mañes S (2013). CX3CL1 at the crossroad of EGF signals: Relevance for the progression of ERBB2(+) breast carcinoma. Oncoimmunology.

[114] Lee Y, Chittezhath M, André V, Zhao H, Poidinger M, Biondi A (2012). Protumoral role of monocytes in human B-cell precursor acute lymphoblastic leukemia: Involvement of the chemokine CXCL10. Blood.

[115] Shin SY, Nam JS, Lim Y, Lee YH (2010). TNFα-exposed bone marrow-derived mesenchymal stem cells promote locomotion of MDA-MB-231 breast cancer cells through transcriptional activation of CXCR3 ligand chemokines. Journal of Biological Chemistry.

[116] Zipin-Roitman A, Meshel T, Sagi-Assif O, Shalmon B, Avivi C, Pfeffer RM, Witz IP, Ben-Baruch A (2007). CXCL10 promotes invasion-related properties in human colorectal carcinoma cells. Cancer Research.

[117] Pellegrino A, Antonaci F, Russo F, Merchionne F, Ribatti D, Vacca A (2004). CXCR3-binding chemokines in multiple myeloma. Cancer Letters.

[118] Zhou H, Wu J, Wang T, Zhang X, Liu D (2016). CXCL10/CXCR3 axis promotes the invasion of gastric cancer via PI3K/AKT pathway-dependent MMPs production. Biomedicine & Pharmacotherapy = Biomedecine & Pharmacotherapie.

[119] Van den Steen PE, Husson SJ, Proost P, Van Damme J, Opdenakker G (2003). Carboxyterminal cleavage of the chemokines MIG and IP-10 by gelatinase B and neutrophil collagenase. Biochemical and Biophysical Research Communications.

[120] Cox JH, Dean RA, Roberts CR, Overall CM (2008). Matrix metalloproteinase processing of CXCL11/I-TAC results in loss of chemoattractant activity and altered glycosaminoglycan binding. The Journal of Biological Chemistry.

[121] Robinson LA, Nataraj C, Thomas DW, Cosby JM, Griffiths R, Bautch VL, Patel DD, Coffman TM (2003). The chemokine CX3CL1 regulates NK cell activity in vivo. Cellular Immunology.

[122] Repnik U, Starr AE, Overall CM, Turk B (2015). Cysteine cathepsins activate ELR chemokines and inactivate non-ELR chemokines. The Journal of Biological Chemistry.

[123] Proost P, Schutyser E, Menten P, Struyf S, Wuyts A, Opdenakker G (2001). Amino-terminal truncation of CXCR3 agonists impairs receptor signaling and lymphocyte chemotaxis, while preserving antiangiogenic properties. Blood.

[124] Liao F (1995). Human Mig chemokine: biochemical and functional characterization. Journal of Experimental Medicine.

[125] Hensbergen PJ, Verzijl D, Balog CIA, Dijkman R, van der Schors RC, van der Raaij-Helmer EMH, van der Plas MJA, Leurs R, Deelder AM, Smit MJ, Tensen CP (2004). Furin is a chemokine-modifying enzyme. Journal of Biological Chemistry.

[126] Ludwig A, Schiemann F, Mentlein R, Lindner B, Brandt E (2002). Dipeptidyl peptidase IV (CD26) on T cells cleaves the CXC chemokine CXCL11 (I-TAC) and abolishes the stimulating but not the desensitizing potential of the chemokine. Journal of Leukocyte Biology.

[127] Inoue A, Hasegawa H, Kohno M, Ito MR, Terada M, Imai T, Yoshie O, Nose M, Fujita S (2005). Antagonist of fractalkine (CX3CL1) delays the initiation and ameliorates the progression of lupus nephritis in MRL/lpr mice. Arthritis and Rheumatism.

[128] Hensbergen PJ, Verzijl D, Balog CIA, Dijkman R, van der Schors RC, van der Raaij-Helmer EMH, van der Plas MJA, Leurs R, Deelder AM, Smit MJ, Tensen CP (2004). Furin is a chemokine-modifying enzyme: in vitro and in vivo processing of CXCL10 generates a C-terminally truncated chemokine retaining full activity. The Journal of Biological Chemistry.

[129] Decalf J, Tarbell KV, Casrouge A, Price JD, Linder G, Mottez E (2016). Inhibition of DPP4 activity in humans establishes its in vivo role in CXCL10 post-translational modification: Prospective placebo-controlled clinical studies. EMBO Molecular Medicine.

[130] Ajami K, Pitman MR, Wilson CH, Park J, Menz RI, Starr AE, Cox JH, Abbott CA, Overall CM, Gorrell MD (2008). Stromal cell-derived factors 1alpha and 1beta, inflammatory protein-10 and interferon-inducible T cell chemo-attractant are novel substrates of dipeptidyl peptidase 8. FEBS Letters.

[131] Zhang H, Maqsudi S, Rainczuk A, Duffield N, Lawrence J, Keane FM, Justa-Schuch D, Geiss-Friedlander R, Gorrell MD, Stephens AN (2015). Identification of novel dipeptidyl peptidase 9 substrates by two-dimensional differential in-gel electrophoresis. FEBS Journal.

[132] Proost P, Mortier A, Loos T, Vandercappellen J, Gouwy M, Ronsse I, Schutyser E, Put W, Parmentier M, Struyf S, van Damme J (2007). Proteolytic processing of CXCL11 by CD13/aminopeptidase N impairs CXCR3 and CXCR7 binding and signaling and reduces lymphocyte and endothelial cell migration. Blood.

[133] Hundhausen C, Schulte A, Schulz B, Andrzejewski MG, Schwarz N, von Hundelshausen P (2007). Regulated shedding of transmembrane chemokines by the disintegrin and metalloproteinase 10 facilitates detachment of adherent leukocytes. Journal of Immunology (Baltimore, Md. : 1950).

[134] Garton KJ, Gough PJ, Blobel CP, Murphy G, Greaves DR, Dempsey PJ (2001). Tumor necrosis factor-alpha-converting enzyme (ADAM17) mediates the cleavage and shedding of fractalkine (CX3CL1). The Journal of Biological Chemistry.

[135] Ludwig A, Weber C (2007). Transmembrane chemokines: versatile “special agents” in vascular inflammation. Thrombosis and Haemostasis.

[136] Johnson LA, Jackson DG (2013). The chemokine CX3CL1 promotes trafficking of dendritic cells through inflamed lymphatics. Journal of Cell Science.

[137] Bourd-Boittin K, Basset L, Bonnier D, L’Helgoualc’h A, Samson M, Théret N (2009). CX3CL1/fractalkine shedding by human hepatic stellate cells: Contribution to chronic inflammation in the liver. Journal of Cellular and Molecular Medicine.

[138] Malcangio M, Clark AK (2012). Microglial signalling mechanisms: cathepsin S and fractalkine. Experimental Neurology.

[139] Wildenberg ME, van Helden-Meeuwsen CG, Drexhage HA, Versnel MA (2008). Altered fractalkine cleavage potentially promotes local inflammation in NOD salivary gland. Arthritis Research and Therapy.

[140] Juric V, O’Sullivan C, Stefanutti E, Kovalenko M, Greenstein A, Barry-Hamilton V (2018). MMP-9 inhibition promotes anti-tumor immunity through disruption of biochemical and physical barriers to T-cell trafficking to tumors. PLoS One.

[141] Nishina S, Yamauchi A, Kawaguchi T, Kaku K, Goto M, Sasaki K, Hara Y, Tomiyama Y, Kuribayashi F, Torimura T, Hino K (2019). Dipeptidyl peptidase 4 inhibitors reduce hepatocellular carcinoma by activating lymphocyte chemotaxis in mice. Cellular and Molecular Gastroenterology and Hepatology.

[142] Hollande C, Boussier J, Ziai J, Nozawa T, Bondet V, Phung W, Lu B, Duffy D, Paradis V, Mallet V, Eberl G, Sandoval W, Schartner JM, Pol S, Barreira da Silva R, Albert ML (2019). Inhibition of the dipeptidyl peptidase DPP4 (CD26) reveals IL-33-dependent eosinophil-mediated control of tumor growth. Nature Immunology.

[143] Rainczuk A, Rao JR, Gathercole JL, Fairweather NJ, Chu S, Masadah R, Jobling TW, Deb-Choudhury S, Dyer J, Stephens AN (2014). Evidence for the antagonistic form of CXC-motif chemokine CXCL10 in serous epithelial ovarian tumours. International Journal of Cancer.

